# Postmitotic Prox1 Expression Controls the Final Specification of Cortical VIP Interneuron Subtypes

**DOI:** 10.1523/JNEUROSCI.1021-21.2021

**Published:** 2021-09-29

**Authors:** Tevye Jason Stachniak, Rahel Kastli, Olivia Hanley, Ali Özgür Argunsah, Elianne Grietje Theodora van der Valk, George Kanatouris, Theofanis Karayannis

**Affiliations:** ^1^Laboratory of Neural Circuit Assembly, Brain Research Institute, University of Zurich, CH-8057, Zurich, Switzerland; ^2^Neuroscience Center Zurich, Zurich, CH-8057, Switzerland

**Keywords:** developmental biology, Elfn1, interneuron, Prox1, synapse, VIP

## Abstract

Throughout development, neuronal identity is controlled by key transcription factors that determine the unique properties of a cell. During embryogenesis, the transcription factor Prox1 regulates VIP-positive cortical interneuron migration, survival, and connectivity. Here, we explore the role of Prox1 as a regulator of genetic programs that guide the final specification of VIP interneuron subtypes in early postnatal life. Synaptic *in vitro* electrophysiology in male and female mice shows that postnatal Prox1 removal differentially affects the dynamics of excitatory inputs onto VIP bipolar and multipolar subtypes. RNA sequencing reveals that one of the downstream targets of Prox1 is the postsynaptic protein Elfn1, a constitutive regulator of presynaptic release probability. Further genetic, pharmacological, and electrophysiological experiments demonstrate that removing Prox1 reduces Elfn1 function in VIP multipolar but not in bipolar cells. Finally, overexpression experiments and analysis of native *Elfn1* mRNA expression reveal that Elfn1 levels are differentially controlled at the post-transcriptional stage. Thus, in addition to activity-dependent processes that contribute to the developmental trajectory of VIP cells, genetic programs engaged by Prox1 control the final differentiation of multipolar and bipolar subtypes.

**SIGNIFICANCE STATEMENT** The transcription factor Prox1 generates functional diversification of cortical VIP interneuron subtypes in early postnatal life, thus expanding the inhibitory repertoire of the cortex.

## Introduction

Although cortical interneuron (IN) diversity begins at their place of birth within distinct embryonic progenitor domains (Mi et al., 2018), single-cell sequencing and manipulation experiments at different developmental stages have suggested that INs undergo their final specification while incorporating into the developing circuit (De Marco García et al., 2011; Mayer et al., 2018). The developmental mechanisms by which distinct types of INs acquire their mature characteristics are only beginning to be revealed (Favuzzi et al., 2019). Interestingly, postmitotic manipulations have demonstrated a persistent requirement for key transcription factors (TFs) in the final specification and maintenance of pyramidal cell fate (De La Rossa et al., 2013). Whether similar TF mechanisms exist for cortical INs remains unknown.

Cortical vasoactive intestinal peptide-expressing (VIP) INs are a diverse population that can be roughly classified into two subgroups: the bipolar and multipolar VIP cells (Bayraktar et al., 1997, 2000; Cauli et al., 2014; Prönneke et al., 2015; Tasic et al., 2018). Although they make up <5% of all neurons, VIP INs are critically important for cortical circuit maturation and their malfunction has been implicated in neurodevelopmental disorders (Batista-Brito et al., 2017; Mossner et al., 2020). The TF Prospero-related homeobox1 (Prox1) is expressed by the majority of INs derived from the caudal ganglionic eminence (Rubin and Kessaris, 2013; Cauli et al., 2014). Prox1 removal during embryonic development impairs VIP cell migration, cell survival, as well as dendritic development and afferent connectivity of the calretinin-expressing (CR^+^) VIP bipolar subtype (Miyoshi et al., 2015). Importantly, Prox1 remains expressed in all VIP INs as they incorporate into the developing circuit, suggesting it may have a role in organizing not only their early stages of development, but also their final specification. In addition to requiring Prox1, it has been shown that CR^+^ VIP bipolar cells also depend on proper network activity to acquire their characteristic axo-dendritic profile, as do Reelin- and somatostatin (SST)-expressing INs (De Marco García et al., 2011, 2015; Pan et al., 2018). In contrast, the cholecystokinin-expressing (CCK^+^) VIP multipolar subtypes do not show any morphological deficits following activity manipulations (De Marco García et al., 2011), and the effect of Prox1 removal has not been well explored in this cells (Miyoshi et al., 2015). Together, these findings raise the possibility that, during the final developmental steps of VIP IN specification, cell-autonomous and activity-dependent genetic programs work in tandem to guide the network incorporation of VIP subtypes in a differential manner.

In this study, we assess the postnatal requirement of Prox1 for the final diversification of VIP IN subtypes. To achieve this, we use a *VIPCre* driver mouse line combined with a conditional *Prox1* allele to remove this TF during the first postnatal week (Prox1 postnatal conditional knockout [cKO]) and explore the synaptic properties of bipolar and multipolar VIP cells. Surprisingly, we find that postnatal Prox1 removal leads to an alteration of the short-term synaptic dynamics of incoming excitation onto VIP multipolar but not bipolar cells. Using a transcriptomic screen and pharmacological manipulations, we demonstrate that a Prox1-dependent engagement of the trans-synaptic protein Extracellular Leucine Rich Repeat and Fibronectin type III Domain Containing 1 (Elfn1) selectively enables the stronger synaptic facilitation observed in multipolar cells.

## Materials and Methods

### 

#### Mice

All animal experiments were approved by the Cantonal Veterinary Office Zurich and followed Swiss national regulations. Animal lines used in this study are as follows: *VIPCre* (Vip^tm1(cre)Zjh/J^) (Taniguchi et al., 2011), *Ai14* (B6;129S6-Gt(ROSA)^26Sortm14(CAG-tdTomato)Hze/J^) (Madisen et al., 2010), *Prox1fl* (Prox1^tm2Gco^) (Harvey et al., 2005), *Prox1eGFP* (Prox1^tm1.1Fuma^) (Iwano et al., 2012), and *Elfn1KO* (Elfn1^tm1(KOMP)Vlcg^) (created by the Knock Out Mouse Project). The following compound lines were created for this study: *VIPCre x Prox1eGFP* and *VIPCre x Ai14 x Elfn1KO* for staining and electrophysiology experiments; and *VIPCre x Ai14 x Prox1fl* for mGluR7 staining, *in situ*, and Elfn1 overexpression experiments. All crosses were set up to produce both KO and control animals in the same litter (using both males and females), and littermate controls were used throughout the study.

#### Electrophysiology

Whole-cell patch-clamp electrophysiological recordings were performed on fluorescently-labeled VIP neurons located in neocortical layers 2 and 3 of barrel cortex (L2/3, approximately bregma −0.5 to −2.0 mm) in acute brain slices prepared from postnatal day 17-34 (P17-P34) male and female mice. Briefly, animals were decapitated, and the brain was dissected out and transferred to cold cutting solution containing the following (in mm): 75 sucrose, 87 NaCl, 25 NaHCO_3_, 25 D-glucose, 2.5 KCl, 7 MgCl_2_, and 1.25 NaH_2_PO_4_, aerated with 95% O_2_/5% CO_2_; 300 μm slices were recovered in ACSF composed of the following (in mm): 128 NaCl, 3 KCl, 26 NaHCO_3_, 1.25 NaH_2_PO_4_, 1 MgCl_2_, 2 CaCl_2_, and 10 glucose at 34°C for 15 min. Acute slices were perfused at a rate of 2-3 ml/min with oxygenated recording ACSF at room temperature. Patch electrodes were made from borosilicate glass (Harvard Apparatus) and had a resistance of 2-4 mΩ. The intracellular solution contained the following (in mm): 126 cesium methanesulfonate, 4 CsCl, 10 HEPES, 20 phosphocreatine, 4 MgATP, 0.3 NaGTP, pH 7.3, 290 mOsm, with addition of 2.5 mg/ml of biocytin.

Experiments were performed in voltage-clamp mode using the Axopatch 200B amplifier (Molecular Devices). Visually guided patch of fluorescently-labeled cells was performed on a Carl Zeiss Axioscope using a Retiga Electro camera (01-ELECTRO-M-14-C-OC, Teledyne Scientific Imaging). Access resistance was monitored to ensure the stability of recording conditions. Recordings with access resistance >40 mΩ, or whole-cell capacitance <4 pF were excluded. No compensation was made for access resistance, and no correction was made for the junction potential between the pipette and the ACSF. Following a baseline stabilization period (2-3 min), evoked synaptic currents recorded in 2 min (12 sweeps) at V_h_= −70 mV were averaged and analyzed using Clampfit10 (Molecular Devices). Two electrical stimuli from a Digitimer isolated stimulator (DS2A Mk.II) were delivered at 50 Hz through a monopolar glass pipette (2-4 mΩ) positioned in L2/3, close to the soma or proximal dendrites of the recorded cells. The stimulating electrode was placed typically 50-150 μm from the recorded cell, parallel to the pial surface. Stimulation intensity and duration were adjusted to produce stable evoked EPSC amplitudes. Stimulation intensities were larger for bipolar cells (86 ± 1 V for 103 ± 6 µs) than for multipolar cells (74 ± 2 V for 86 ± 6 µs, *p* = 3 × 10^−6^, *p* = 0.06) but did not differ between genotypes (multipolar Prox1 control: 69 ± 3 V for 57 ± 5 µs, cKO: 71 ± 4 V for 74 ± 10 µs, *p* = 0.6, *p* = 0.2; bipolar Prox1 control: 85 ± 2 V for 91 ± 9 µs, cKO: 84 ± 3 V for 88 ± 15 µs, *p* = 0.9, *p* = 0.9; multipolar Elfn1 control: 77 ± 3 V for 114 ± 13 µs, Het: 82 ± 3 V for 125 ± 19 µs, *p* = 0.2, *p* = 0.6; bipolar Elfn1 control: 86 ± 3 V for 113 ± 13 µs, Het: 89 ± 2 V for 130 ± 15 µs, *p* = 0.3, *p* = 0.4, *t* test). For comparison between L2/3 and L4 stimulation, the stimulating electrode was moved from the initial position in L2/3 to L4, parallel to the bipolar cell dendrites. Stimulation intensity and duration were held constant (61 ± 4 V for 63 ± 5 µs). (RS)-α-methylserine-O-phosphate (MSOP; 100 μm, pH 7.4) was dissolved in water and applied in the bath.

#### Cell sorting and RNA sequencing

eGFP-labeled cells were purified from the P12 cortices of control and cKO Prox1 animals *(VIPCre x Prox1eGFP*/+ and *VIPCre x Prox1eGFP/eGFP*) as described previously (Hempel et al., 2007). Briefly, animals were anesthetized in 2% v/v isoflurane (∼1.5 min), decapitated, the brain was extracted, the olfactory bulb and cerebellum were removed, and the brain was cut into 400-μm-thick coronal sections using a vibratome (Leica Microsystems, VT1000S; speed 5, vibration frequency 7-8), while in bubbling ice-cold ACSF (recipe as above, with 1 mm CaCl_2_ and 1 mm MgCl_2_) in the chamber. Sections were collected and transferred into a protease digestion solution of ACSF (with Pronase, 1 mg ml^−1^), for 25 min and then transferred into a quenching solution of ACSF (with 1% FBS). Microdissection of the cortex to include the somatosensory areas was performed using a fluorescent dissection microscope (Olympus, MVX 10). Dissected cortices were collected in a 15 ml Falcon tube containing 1.5 ml of ACSF sorting solution containing 1% FBS and DNase, and cells were dissociated by gently triturating 10 times with a large, then medium, and finally small fire-polished Pasteur pipette while avoiding the generation of bubbles. The cell suspension was then passed through a 50 μm filter (Sysmex CellTrics) before automated FACS using the MoFlo or FACSAria devices. A GFP-negative littermate control cortex was also included as a negative control for the FACS setup. Cells were collected into Arcturus Picopure extraction buffer and immediately processed for RNA isolation using the Arcturus PicoPure Isolation Kit (KIT0204). RNA quality and quantity were measured using Agilent High Sensitivity RNA ScreenTape system (High Sensitivity RNA Screen Tape 5067-5579-5580-5581). All samples had high-quality scores between 6 and 8 RIN. Four or five RNA samples of each genotype were used to prepare 19 barcoded libraries.

The libraries were prepared using the Smart-seq2 protocol (Picelli et al., 2013). Briefly, total RNA was placed in 4 µl of lysis buffer (0.1% v/v Triton X-100, 2.5 mm dNTPs, 2.5 μm oligo-dT, 1 U/µl Promega Rnasin Plus RNase inhibitor). Reverse transcription was performed followed by cDNA amplification. The quality of the cDNAs was evaluated using an Agilent 2100 Bioanalyzer. The resulting cDNA (1 ng) was fragmented using Illumina Nextera XT according to standard protocol. Nextera adaptors containing Unique Dual Indices were added by PCR. The libraries were double-sided size, selected, and quantified using an Agilent 4200 TapeStation System.

TruSeq SR Cluster Kit HS4000 (Illumina) was used for cluster generation using 10 pm of pooled normalized libraries on the cBOT. Sequencing was performed on the Illumina HiSeq 4000 single end 100 bp, using the TruSeq SBS Kit HS4000 (Illumina).

#### Prox1 stainings

*VIPCre x Prox1eGFP* (Control and cKO) animals age P4 and P6 were deeply anesthetized before transcardial perfusion with first 1× PBS followed by ice-cold 4% PFA. The brains were dissected and postfixed in ice-cold 4% PFA for 1 h, then cryo-protected in a 30% sucrose solution at 4°C for >24 h. The brains were embedded in OCT using a peel-away mold and stored at −80°C. Coronal 20-μm-think brain sections containing barrel cortex were cut and collected on-slide using a cryostat (Microm International, HM 560 M), and the slides were stored at −80°C until further processing.

The slides were thawed and washed using 1× PBS (3× 5min) before being blocked using 1.5% normal donkey serum (NDS) and 0.05% Triton X-100 in 1× PBS for 1 h. The primary antibodies GFP (chicken anti GFP, Abcam, ab13970) and Prox1 (goat anti Prox1, R&D Systems, AF2727) were both diluted 1:1000 in the blocking solution and left to incubate overnight at 4°C. The next day, the slides were washed (3× 7min) with 1× PBS before applying the secondary antibodies (donkey anti-goat 650, Thermo Fisher Scientific, SA5-10089 and donkey anti-chicken, Jackson ImmunoResearch Laboratories, 703-545-155) diluted at 1:1000 in 1× PBS for 2 h. The slides were coverslipped with Fluoromount-G with DAPI (00-4959-52) and imaged using an inverted Leica Microsystems SP8 microscope.

#### mGluR7 staining

*VIPCre x Ai14 x Prox1fl* (Control and cKO) animals aged P21 were perfused, postfixed, and cut at 20 µm as described above. Cryosections were washed in 0.3% Triton X-100 in 1× PBS (3× 5 min) and blocked for 2 h using 5% BSA, 10% NDS, 0.3% Triton X-100 in 1× PBS. The primary antibodies mGluR7 (rabbit anti mGluR7, Millipore, 07-239) and tdTomato (goat anti-tdTomato, SICGEN Antibodies, AB8181-200) were diluted at 1:500 and 1:1000 in 1% BSA, 5% NDS, 0.3% Triton X-100 in 1× PBS and applied to the slide overnight at 4°C. The next day, the slides were washed (4 × 7 min) with 1× PBS before applying the secondary antibody solution containing donkey anti-rabbit 488 (Thermo Fisher Scientific, A21206) and donkey anti-goat Cy3 (Jackson ImmunoResearch Laboratories, 705-165-147) diluted at 1:1000 in the same solution as the primary antibodies and left to incubate for 2 h at room temperature. They were then coverslipped using Vectashield with DAPI (H-1200-10) and stored at 4°C until imaging.

Images were taken on a Leica Microsystems SP8 inverted microscope, with a 63× oil immersion objective at 1× zoom. Upper L2/3 and L4 were identified using the DAPI signal, and images sized 1600 × 1600 pixels were taken in both locations with voxel size 0.1154 × 0.1154 × 0.297 μm (*XYZ*).

#### mRNA ISH experiments

*VIPCre* x *Ai14* x *Prox1fl* animals aged P12 were perfused, postfixed, and cut at 20 µm as described above. ISH for *Elfn1* mRNA and *CR (Calb2)* mRNA was performed using the RNAscope kit (RNAscope Intro Pack for Multiplex Fluorescent Reagent Kit v2- Mm, 323136). Briefly, the slides were thawed, and OCT residue was removed using 1× PBS (3 × 5 min washes). The slides were then baked for 30 min at 60°C, postfixed for 30 min in 4% PFA, dehydrated in an Ethanol dilution series (50%, 70%, 2 × 100%), and incubated with RNAscope Hydrogen Peroxide for 10 min. RNAscope 1× Target Retrieval Reagents was brought to the boil, and the slides were submerged for 2 min. Protease III was added to the slides and left to incubate for 45 min at 40°C. After the pretreatment, the probes (*CR*: Mm-Calb2, 313641 and *Elfn1*: Mn-Elfn1, 449661-C3) were hybridized to the slices for 2 h at 40°C, and the signal was amplified using branched DNA amplification methods and visualized with Opal dyes (Opal 520 FP1487001KT and Opal690 FP1497001KT).

Following the RNAscope protocol, the slices were immunostained to retrieve the tdTomato signal that was lost during the RNAscope protocol. Slides were washed 2 times with 1× PBS for 5 min before being blocked for 30 min using 10% NDS and 1% BSA in 1× PBS. The primary antibody against tdTomato (goat anti tdTomato, SICGEN Antibodies, AB8181-200) was diluted 1:700 in 1× PBS 1% BSA and left to incubate at 4°C overnight followed by a 2 h incubation of secondary antibody at room temperature (donkey anti-goat Cy3, Jackson ImmunoResearch Laboratories, 705-165-147, diluted 1:1000 in 1× PBS 1% BSA). Slides were coverslipped with Fluoromount-G with DAPI (00-4959-52) and imaged using a Slidescanner (Carl Zeiss, AxioScan Z1). Mosaic images of the whole cortex were taken using a 20× objective.

#### Elfn1 overexpression/rescue experiments

To induce cell type-selective Elfn1 overexpression, a custom AAV plasmid was created using a three-way ligation of the 4.4 kb KpnI/BsrGI-fragment of the Addgene #28305 plasmid (backbone-ITRs-Promoter-dlox sites), the 0.8 kb BsrGI/BamHI-fragment of the Addgene #85225 plasmid (2A-EGFP), and a 2.5 kb PCRfragment from an Elfn1-GFP plasmid (*Elfn1* sequence) (Sylwestrak and Ghosh, 2012). The custom virus is available on request from the viral vector facility at the University of Zurich (https://www.vvf.uzh.ch/).

*VIPCre x Ai14 x Prox1fl* control and cKO pups were injected with the Elfn1-AAV construct at P4. They were anesthetized using isoflurane, and the injection was performed with a glass micropipette attached to a Nanolitre 2010 pressure injection apparatus (World Precision Instruments). The micropipette was inserted into the barrel field, and a total of 210 nl (70 nl each) of virus was injected at three different depths (300, 200, and 100 µm). The pups were then placed back in their home-cage and monitored for 4 d. Two to 4 weeks after the injection, they were killed and used for electrophysiology recordings as described above.

#### Experimental design and statistical analysis

##### Electrophysiology data analysis

Values are represented as mean ± SEM. Number of measurements (*n*/*N*) indicates cells recorded (*n*) from animals (*N*), typically using 1 cell per slice to recover biocytin-stained cell morphology. Cell type was classified as bipolar versus multipolar based on cell body morphology (ovoid vs round) and number and orientation of dendritic processes emanating from it (2 or 3 dendrites perpendicular to pia (for bipolar) versus ≥3 processes in diverse orientations (for multipolar) (for a more comprehensive description, see also Cauli et al., 2014). In addition, the layer localization of the two populations differed, with multipolar cells found primarily in the upper L2, compared with the bipolar cells found throughout L2/3. Classification used both the initial determination, before patching fluorescent-labeled cells, and *post hoc* verification of recovered biocytin-labeled cells. Matched recordings were performed with Prox1 control and Prox1 cKO littermates on the same day, whenever possible. Elfn1 rescue experiments included control recordings from uninfected neighboring cells in tissue transfected with Elfn1-GFP virus. Elfn1-GFP transfection of patched cells was verified by measuring green fluorescence intensity (average fluorescence intensity > 6000 a.u.). Statistical testing was done in MATLAB. Comparisons within conditions were made by two-tailed paired Student's *t* test, treatment versus baseline. Comparisons across conditions or between genotypes were done with an unpaired *t* test assuming unequal variance. For multiple comparisons, a one-way or two-way ANOVA was done with a Bonferroni *post hoc* test.

##### RNA sequencing data analysis

The raw sequence reads were first cleaned by removing adapter sequences, trimming low-quality ends, and filtering reads with low quality (phred quality <20) using Trimmomatic (version 0.33) (Bolger et al., 2014). The read alignment was done with STAR (version 2.5.3a) (Dobin et al., 2013) As a reference, we used the Ensembl genome build GRCm38 with the gene annotations downloaded on 2015-06-25 from Ensembl. The STAR alignment options were as follows: “–outFilterType BySJout –outFilterMatchNmin 30 –outFilterMismatchNmax 10 –outFilterMismatchNoverLmax 0.05 –alignSJDBoverhangMin 1 –alignSJoverhangMin 8 –alignIntronMax 1000000 –alignMatesGapMax 1000000 –outFilter MultimapNmax 50.”

Gene expression values were computed with the function featureCounts from the R package Rsubread (version 1.26.0) (Liao et al., 2013). The options for featureCounts were as follows: min mapping quality 10 – min feature overlap 10 bp – count multimapping reads – count only primary alignments – count reads also if they overlap multiple genes. One sample was excluded from further analysis based on quality control standards (see Fig. 2*B*).

To detect differentially expressed genes, we applied a count-based negative binomial model implemented in the software package EdgeR (R version: 3.6.0, EdgeR version: 3.26.1) (Robinson et al., 2010). The differential expression was assessed using an exact test adapted for overdispersed data. Genes showing altered expression with adjusted (Benjamini and Hochberg method) *p* value <0.05 were considered differentially expressed.

A list of potential candidate genes was generated by selecting genes that had a log2ratio≥|0.5| and *p* value ≤ 0.01 (see Extended Data Fig. 2-1). This list was analyzed for Gene Ontology (GO) enrichment using g:Profiler (Raudvere et al., 2019) (https://biit.cs.ut.ee/gprofiler/gost).

##### mGluR7 staining data analysis

A customized MATLAB script was used to analyze 3D mGluR7 colocalizations on VIP-tdTomato-positive processes. First, intensity histograms of tdTomato signal (channel 1) and mGluR7 signal (channel 2) were normalized to a corresponding reference image. Next, cell somas were removed from the tdTomato channel according to the following pipeline. First, a blob enhancement filter was used by convolving tdTomato channel with a Gaussian kernel to increase the signal-to-noise ratio. After, tdTomato-positive cell bodies were segmented using Otsu thresholding. Active contours were then used to finalize the segmentation. Finally, the masked tdTomato-positive somas were removed from the dataset, leaving exclusively tdTomato-positive VIP processes in channel 1. Otsu thresholding was performed on the histogram-matched channel 2 to segment mGluR7 puncta. Last, 3D masked stacks for channels 1 and 2 were combined with a logical AND operator and 3D connected components analysis was used to count the putative mGluR7 puncta on VIP processes. The number of colocalizations was normalized by the volume of the processes.

##### mRNA ISH data analysis

Image analysis was performed using custom-written MATLAB codes. A two-dimensional difference of Gaussian feature enhancement algorithm was used to improve the VIP-tdTomato images, followed by Otsu thresholding to get an initial segmentation of neuronal cell bodies. To ensure an accurate representation of the cell body, segmentation of VIP INs was finalized using active contour segmentation (Kass et al., 1988).

During preprocessing of the *CR* and *Elfn1* images, background subtraction was performed using a disk-shaped structuring. Subsequently, fluorescent intensity levels of both *CR* and *Elfn1* were measured within each of the segmented VIP IN cell bodies. The somatosensory region, as well as L1-L6, were segmented manually using the DAPI channel as reference (see Fig. 4*A*,*B*). To define whether a VIP cell is CR^+^, we followed a Bayesian approach by assuming 80% of VIP cells are CR^+^ (Kubota et al., 2011). Hence, once mean *CR mRNA* intensity per cell was calculated for each image, the population was thresholded such that ∼80% of total VIP cells are considered CR^+^. Statistical analysis of the data was done using the Mann–Whitney *U* test.

#### Data and code availability

Data and custom-written codes are available on request. The RNAseq data have been uploaded to the ENA database (accession number PRJEB37790).

## Results

### Postnatal Prox1 removal leads to changes in short-term dynamics of incoming excitatory events onto VIP multipolar but not bipolar cells

**Figure 1.**
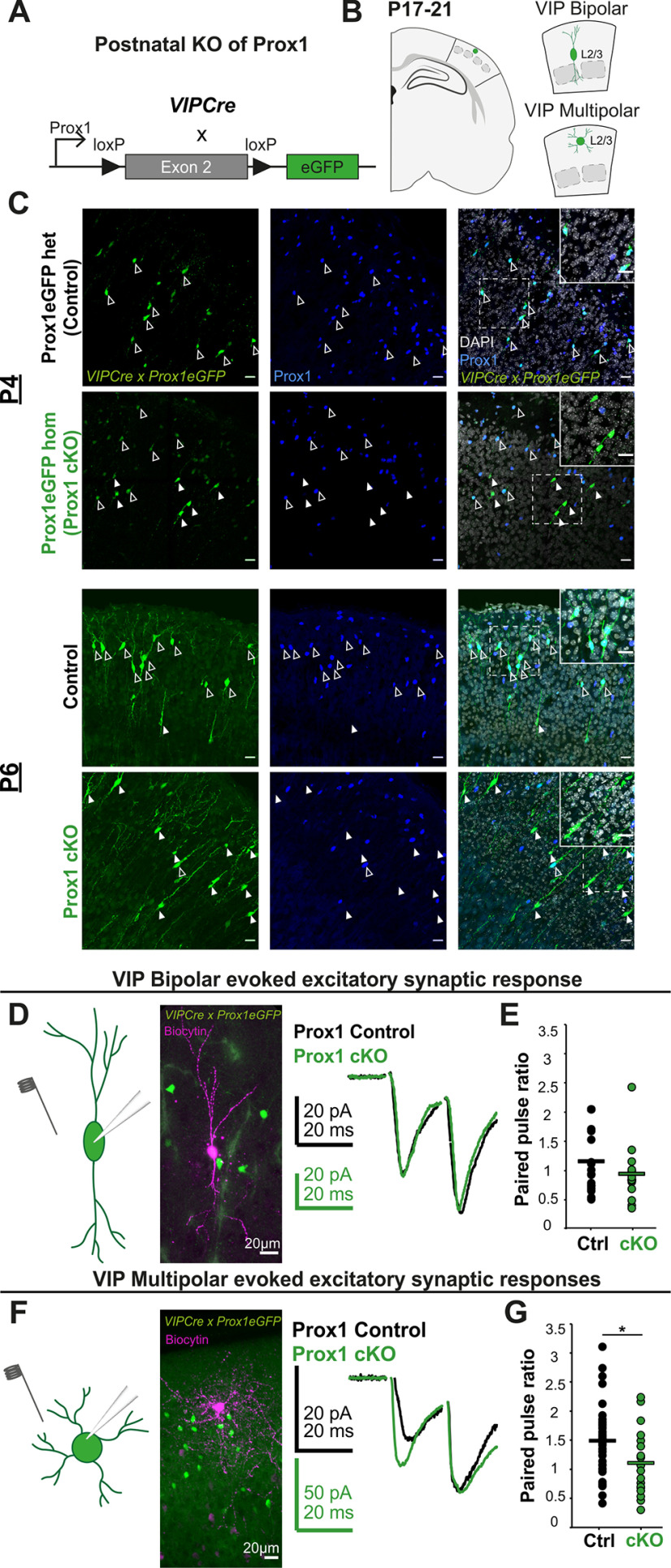
Postnatal Prox1 removal leads to changes in short-term dynamics of incoming excitatory events onto VIP multipolar but not bipolar cells. ***A***, Visual representation of *Prox1* cKO strategy. *VIPCre* turns on postnatally and removes part of the coding region of the *Prox1* locus, shifting eGFP into frame and allowing for the visualization of the control (Prox1 het) and Prox1 cKO cells. ***B***, Schematic representation of the experiment. L2/3 multipolar and bipolar VIP cells in the somatosensory barrel cortex were stained for Prox1 at P4 and P6 (***C***) and recorded at P17-21 in acute brain slices from control and Prox1 cKO animals (***D***). ***C***, Images of Prox1 staining in L2/3 *VIPCre x Prox1eGFP* cells at P4 (top) and P6 (bottom) in control and Prox1 cKO tissue. At P4, recombination of the *Prox1* allele has already begun, and many strongly eGFP-positive cells show no Prox1 labeling. At P6, almost all VIPCre cells are strongly fluorescent, and recombination of the allele is complete as shown by the lack of Prox1 expression in the fate-mapped cells. Open arrows highlight VIP-positive, Prox1-positive cells. Filled arrows indicate VIP-positive, Prox1-negative cells. Insets, Close-up of the dashed-line boxes. ***D***, ***F***, Left, Schematic representation for probing the evoked excitatory synaptic responses onto bipolar and multipolar Prox1 control and cKO VIP cells. The stimulating electrode is shown close to the soma and proximal dendrites of the eGFP-positive cells, which are recorded in whole-cell patch-clamp mode. Middle, The recorded cells were filled with biocytin, and their morphology revealed *post hoc*. Right, Examples of a pair of evoked excitatory synaptic responses for control and cKO cells, which are overlaid and scaled to the second response. ***E***, All data points for PPR (second/first response) for control (*n*/*N* = 18/11) and cKO (*n*/*N* = 13/11) bipolar VIP cells (*p* = 0.3, *t* = 1.1). ***G***, All data points for PPR of control (*n*/*N* = 33/18) and cKO (*n*/*N* = 19/14) multipolar VIP cells (*p* = 0.03, *t* = 2.17). Statistics: *t* test. *p < 0.05.

Cell-specific wiring properties are a prominent feature of IN cell type diversity. Previous research found that embryonic Prox1 removal leads to aberrant network integration of VIP bipolar cells (Miyoshi et al., 2015), whereas the effect on multipolar cells remained unknown. We therefore wanted to examine the effect of postnatal loss of function of Prox1 (cKO) on the synaptic properties of bipolar as well as multipolar VIP cells. To remove Prox1 starting from around P3 onwards, we used a VIP knock-in mouse line that drives the postnatal expression of Cre from the endogenous peptide locus (*VIPCre*) and combined it with a conditional *Prox1* allele. The *Prox1* Exon2 is flanked by *loxp* sites and recombination shifts *eGFP* into frame (*Prox1eGFP)* to label all Prox1-expressing VIP cells (Fig. 1*A*,*B*).

To examine the timeline of Prox1 removal, we collected and sectioned brains from cKO and control *VIPCre x Prox1eGFP* pups at ages P4 and P6 and stained them for GFP and Prox1. At P4, the eGFP signal was weak, but it increased markedly by P6 (Fig. 1*C*), indicating an accumulation of eGFP protein within those 2 d. In line with this result, we observed that at P6 nearly all of the bright eGFP-expressing cKO cells show a complete absence of Prox1 signal, while the majority of control cells display Prox1 signal (Fig. 1*C*). These results show that the removal of Prox1 starts right before P4 and is largely completed by P6.

To assess the involvement of Prox1 in postnatal synaptic properties of VIP subtypes, we examined the short-term dynamics of evoked synaptic responses onto control and Prox1 cKO cells at P17-P21 (Fig. 1*B*). We calculated the paired-pulse ratio (PPR), which provides a stable measure of whether presynaptic release probability for a given synapse is low (PPR > 1), moderate (PPR ∼ 1), or high (PPR < 1). We found that glutamatergic synapses onto control VIP bipolar cells show negligible synaptic facilitation (mean PPR: 1.15 ± 0.13) and that Prox1 cKO does not affect the PPR significantly (mean PPR 0.93 ± 0.15) (Fig. 1*D*,*E*). On the other hand, excitatory inputs onto VIP multipolar cells show more pronounced facilitation in the control condition (mean PPR: 1.51 ± 0.11) and a notable reduction of the PPR on removal of Prox1 (mean PPR: 1.13 ± 0.1) (Fig. 1*F*,*G*). These results demonstrate that, in control conditions, facilitation of glutamatergic synapses onto VIP multipolar cells is higher than onto bipolar cells. Previous reports using paired recordings in the cortex of juvenile or adult animals confirm that ∼80% of pyramidal to bipolar synapses display high release probability with synaptic depression at 18 Hz and that synapses onto L2/3 CR^+^ bipolar cells, on average, display short-term synaptic depression at 10 Hz, with no change at 50 Hz (Porter et al., 1998; Caputi et al., 2009) (https://portal.brain-map.org/explore/connectivity/synaptic-physiology). Importantly, our data also show that postnatal removal of Prox1 leads to a decrease in the facilitation of excitatory synapses onto multipolar, but not bipolar, VIP cells.

### Loss of Prox1 leads to a downregulation of the trans-synaptic protein Elfn1

**Figure 2.**
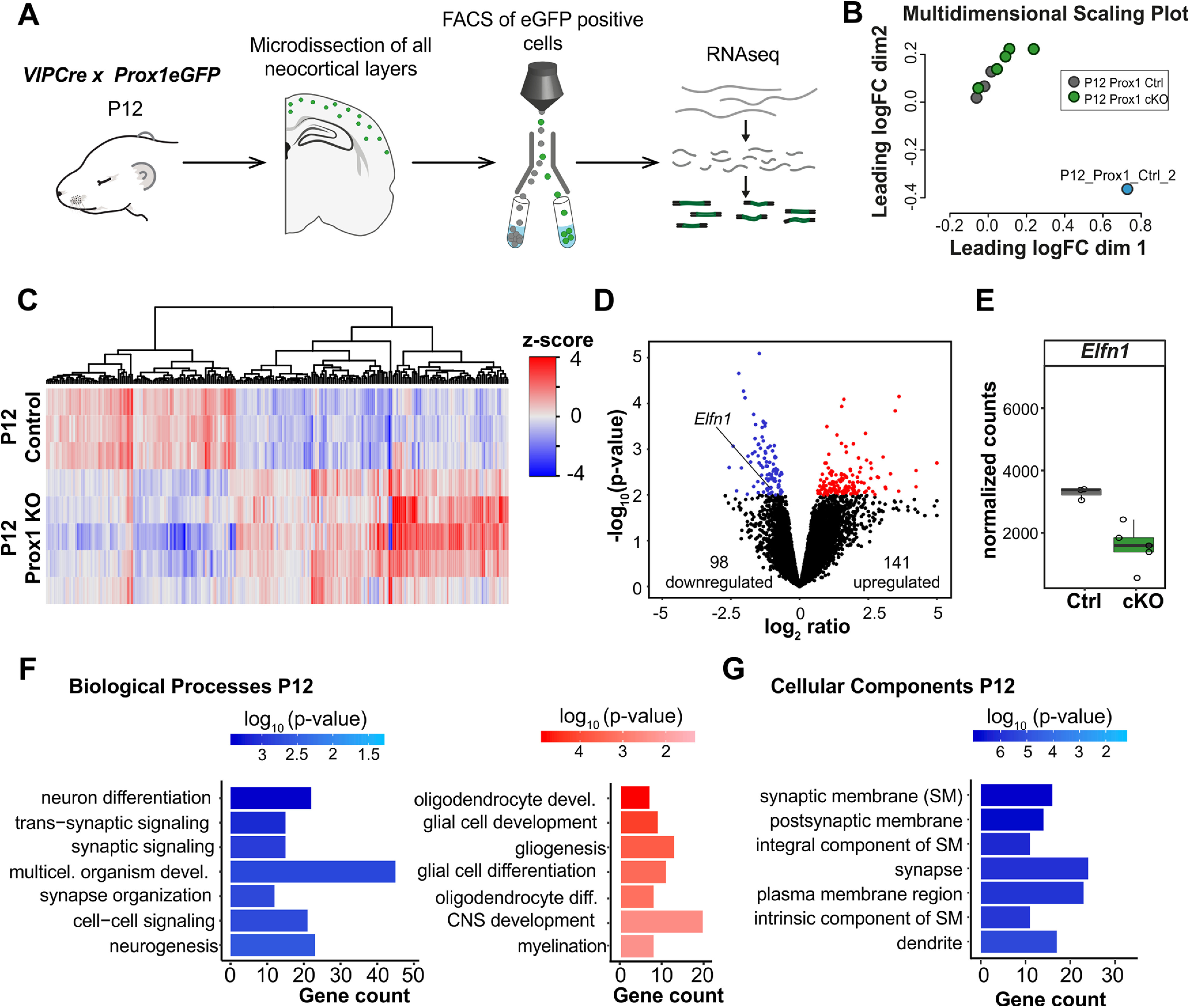
Postnatal removal of Prox1 from VIP cells leads to transcriptomic changes in *Elfn1* and other synapse-related genes. ***A***, Schematic of experimental workflow. *VIPCre x Prox1eGFP* control and cKO cells were sorted using FACS at P8 and P12, and bulk RNA sequencing was performed. ***B***, Multidimensional scaling plot of all the sequenced samples, showing the one control sample that did not meet the quality control criteria and was excluded from the analysis (blue point). ***C***, Heat map showing the clustering according to function of upregulated (red) and downregulated (blue) genes at P12. ***D***, Volcano plot highlighting the differentially expressed candidate genes at P12. They were selected based on log2ratio ≥ |0.5| and *p* value ≤ 0.01. *Elfn1* gene is highlighted. ***E***, *Elfn1* expression levels in P12 control and Prox1 cKO VIP cells. ***F***, GO: Biological Processes enrichment analysis of the candidate genes at P12. Blue represents the downregulated genes. Red represents the upregulated gene. ***G***, GO: Cellular Components enrichment analysis of the candidate genes at P12. Blue represents the downregulated genes. The upregulated genes did not show any clustering in this GO term. See also Extended Data Figure 2-1.

Having identified a Prox1-dependent enhancement of synaptic facilitation onto multipolar cells, we hypothesized that Prox1 transcriptional regulation might impact genes that encode synaptic proteins. To identify such potential downstream targets of Prox1, we performed an RNA sequencing screen on control and Prox1 cKO cells, after FACS of the GFP^+^ VIP cells at P12 (Fig. 2*A*,*B*). Differential gene expression analysis on the data identified several upregulated and downregulated candidate genes (Fig. 2*C*,*D*; see also Extended Data Fig. 2-1), which were analyzed for GO enrichment. The GO analysis revealed that most of the upregulated genes in Prox1 cKO cells are associated with glial cell programs (Fig. 2*F*). This result suggests that, in control VIP cells, Prox1 suppresses those glial cell programs postnatally, directing the cell toward a neuronal fate instead. In line with this, the most enriched downregulated genes are associated with synapses and synapse-associated signaling (Fig. 2*F*,*G*). Within those synapse-associated processes, we specifically looked for genes that could mediate trans-synaptic interactions between the postsynaptic VIP and the presynaptic excitatory cell to regulate synaptic facilitation. One such gene was the *Elfn1* (Fig. 2*D*), which is responsible for the strongly facilitating excitatory synaptic inputs onto SST INs (Sylwestrak and Ghosh, 2012). Postsynaptic Elfn1 produces cell-autonomous suppression of presynaptic glutamate release through its trans-synaptic recruitment of metabotropic-glutamate receptor 7 (mGluR7) (Tomioka et al., 2014; Stachniak et al., 2019).

10.1523/JNEUROSCI.1021-21.2021.f2-1 Figure 2-1 Postnatal Prox1 removal leads to transcriptomic changes in VIP INs. VIP IN candidate genes with a log2ratio≥|0.5| and p value ≤ 0.01 for Prox1 KO versus Prox1 control. Download Figure 2-1, XLSX file.

The RNA sequencing data showed that the level of *Elfn1* mRNA in VIP cells is high at P12 (Fig. 2*E*). To assess whether *Elfn1* is continuously expressed into adulthood, we consulted published single-cell RNA sequencing data (Tasic et al., 2018) and found that, in the adult cortex, almost all VIP cells express *Elfn1* (https://celltypes.brain-map.org/rnaseq/mouse/v1-alm). Furthermore, this dataset also shows a persistent expression of *Prox1*, which suggests a requirement for both these genes in the maintenance of cell function beyond development.

### Reduction in Elfn1 expression recapitulates the Prox1 cKO phenotype in VIP multipolar cells

**Figure 3.**
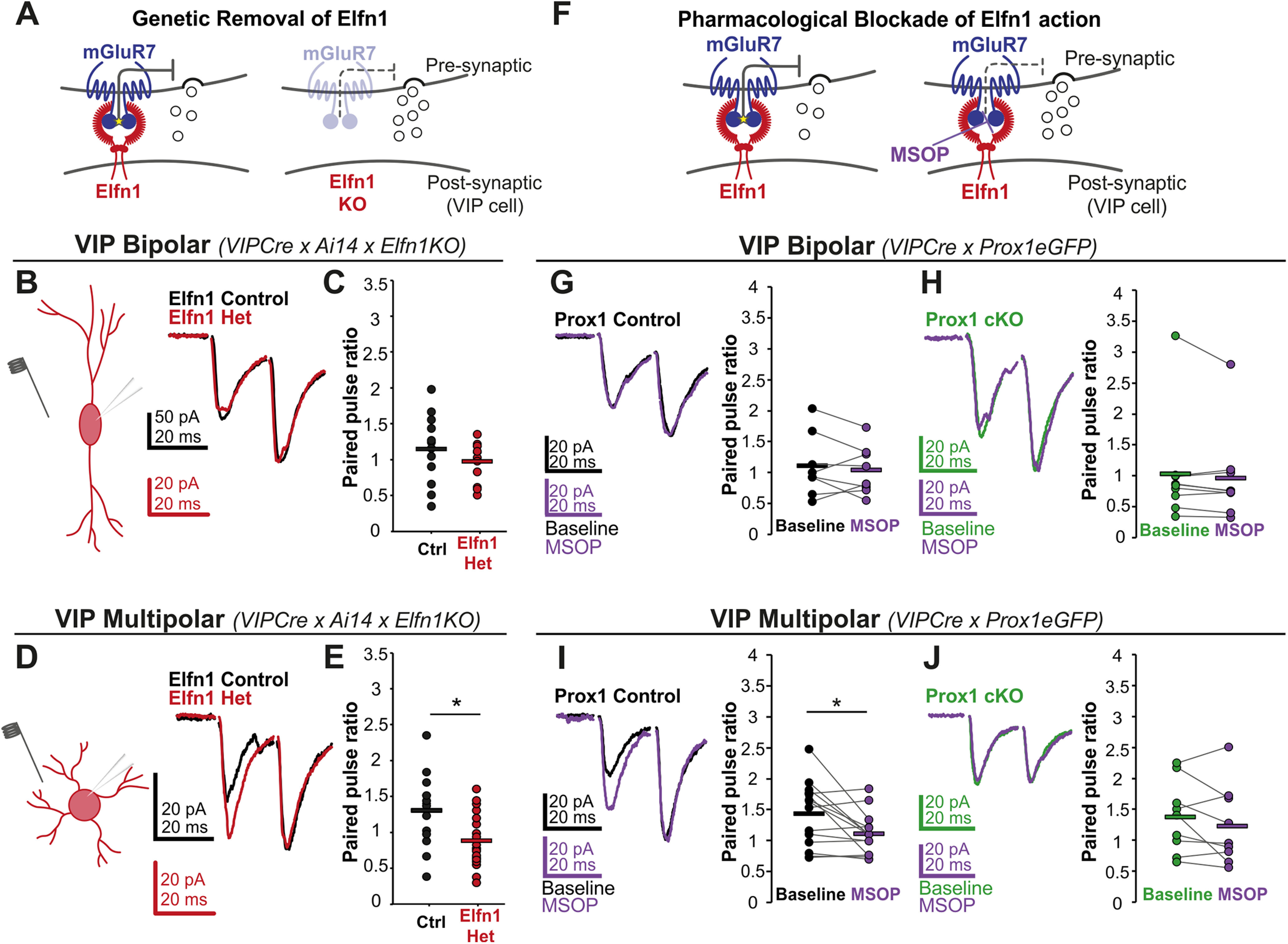
Elfn1 contributes to synaptic facilitation onto VIP multipolar, but not bipolar, cells. ***A***, A compound mouse line labeling VIP cells was crossed into the background of an *Elfn1* germline KO. The presence of Elfn1 decreases initial release probability of glutamate. The experimental and control groups were heterozygous (het) and WT (wt), respectively, for the *Elfn1* germline removal. ***B***, ***D***, Left, Schematic representation of the evoked paired-pulse protocol, with the extracellular stimulating and intracellular recording electrodes shown. Right, Example of a pair of synaptic responses evoked onto VIP bipolar and multipolar cells after stimulation (scaled to second evoked response). ***C***, All data points for PPR of Elfn1 Control (*n*/*N* = 12/4) and Het (*n*/*N* = 12/4) bipolar VIP cells (*p* = 0.3, *t* = 1.06). ***E***, All data points for PPR of Elfn1 Control (*n*/*N* = 14/6) and Het (*n*/*N* = 20/8) multipolar VIP cells (*p* = 0.008, *t* = 2.84). Statistics: *t* test. ***F***, Testing for the effect of MSOP, a presynaptic mGluR blocker and hence for Elfn1 function, on evoked excitatory responses onto control (Het) and Prox1 cKO cells. ***G-J***, Left, Example of a pair of synaptic responses evoked onto VIP bipolar and multipolar cells after stimulation (scaled to second evoked response). Right, All data points for PPR are plotted. ***G***, PPR of bipolar Prox1 Control cells (*n*/*N* = 8/8) plotted before and after the application of MSOP (*p* = 0.5, *t* = 0.74). ***H***, PPR of bipolar Prox1 cKO cells (*n*/*N* = 9/9) plotted before and after the application of MSOP (*p* = 0.2, *t* = 1.26). ***I***, PPR of multipolar Prox1 Control cells (*n*/*N* = 14/9) plotted before and after the application of MSOP (*p* = 0.02, *t* = 2.74). ***J***, PPR of multipolar Prox1 cKO cells (*n*/*N* = 9/8) plotted before and after the application of MSOP (*p* = 0.2, *t* = 1.33). Statistics: paired *t* test. *p < 0.05.

To test whether Prox1 cKO alters the synaptic phenotype of multipolar VIP cells by reducing Elfn1 levels, we used a compound mouse line that labeled VIP neurons (*VIPCre* x tdTomato reporter; *Ai14*) in the background of a germline *Elfn1* KO allele (*Elfn1KO*) (Fig. 3*A*). Since our RNA sequencing screen showed a relatively modest (twofold) reduction of *Elfn1* transcript in Prox1 cKO cortex (Fig. 2*E*), we chose to compare VIP tdTomato cells from heterozygous *Elfn1KO* (Elfn1 Het) animals to those from WT (control) littermates. We found that reducing Elfn1 expression does not affect the PPR significantly in VIP bipolar cells (control mean PPR: 1.15 ± 0.14; Elfn1 Het mean PPR: 0.98 ± 0.08) (Fig. 3*B*,*C*). On the other hand, excitatory inputs onto VIP multipolar cells showed a notable reduction in the PPR when Elfn1 levels are reduced (control mean PPR: 1.30 ± 0.13; Elfn1 Het mean PPR: 0.88 ± 0.08) (Fig. 3*D*,*E*). Our results show that a decrease in Elfn1 expression recapitulates the effect of Prox1 cKO in VIP multipolar cells and has no notable effect in bipolar cells. This finding suggests that Prox1 is important for initiating and/or maintaining the expression of Elfn1 in multipolar cells, which leads to facilitation of incoming excitatory synaptic responses.

### Prox1-dependent engagement of Elfn1 in VIP cells

**Figure 4.**
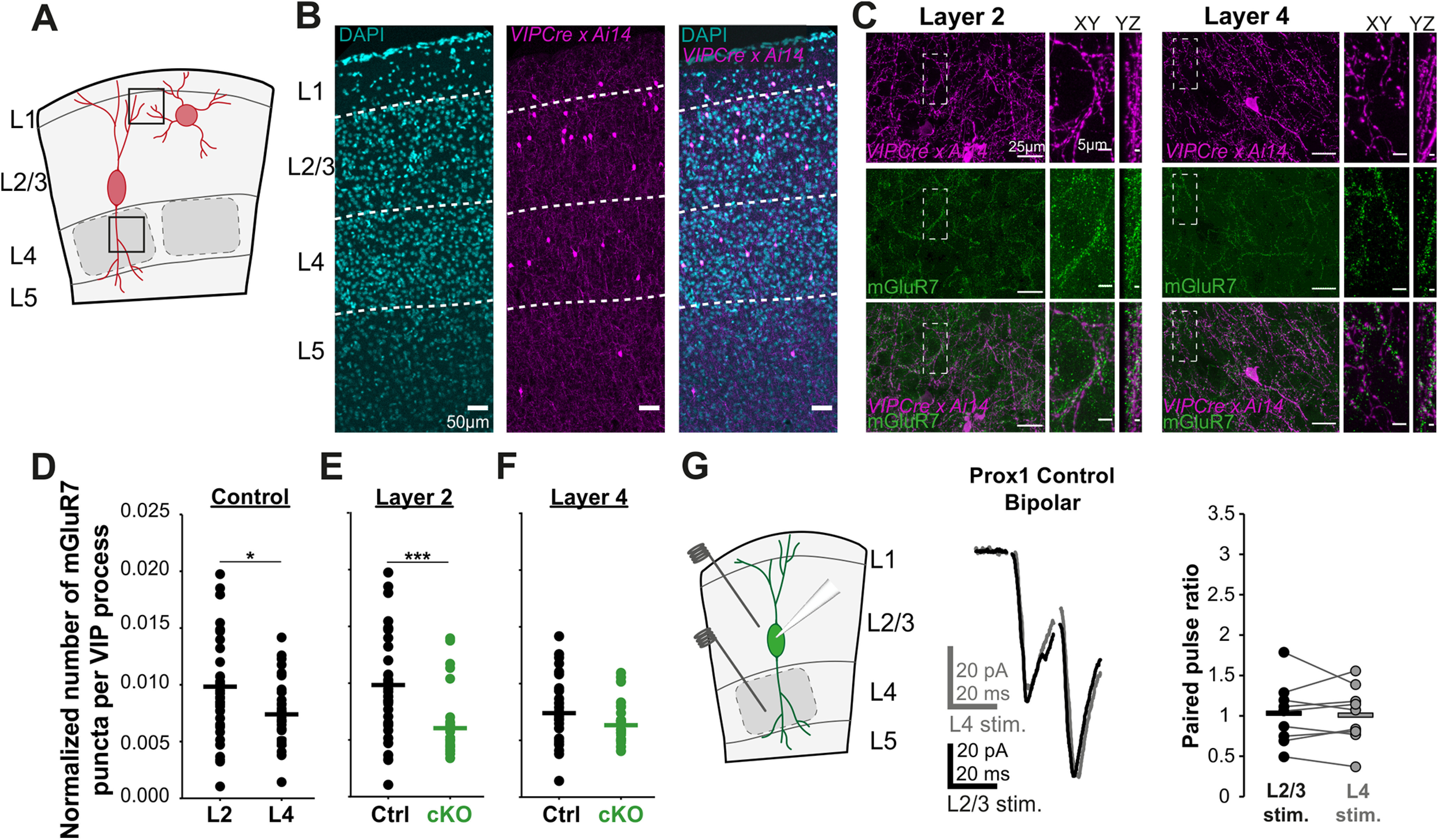
mGluR7 is preferentially recruited to L2 VIP processes. ***A***, Schematic representation of the analysis area in L2/3 containing both multipolar and bipolar processes and in L4 containing only bipolar processes. ***B***, Segmentation of L1-4 in a coronal section of somatosensory cortex based on the DAPI channel. ***C***, Example images of mGluR7 stainings in L2 and L4. Insets, Zoom-in images on the right showing mGluR7 puncta appositions on VIP processes in *xy* and *yz* directions. ***D***, mGluR7 colocalization with VIP processes in L2 and L4 of Prox1 control tissue (*p* = 0.0178) (*n* = 36 images per layer, *N* = 3 animals per genotype). ***E***, mGluR7 colocalization with VIP processes in L2 of control (*n*/*N* = 36/3) and Prox1 cKO (*n*/*N* = 32/3) tissue (*p* = 0.00014). ***F***, mGluR7 colocalization with VIP processes in L4 of control (*n*/*N* = 36/3) and Prox1 cKO (*n*/*N* = 35/3) tissue (*p* = 0.063). Statistics: Mann–Whitney *U* test. ***G***, Left, Schematic illustration of stimulation paradigm for L2/3 bipolar neurons, a stimulation electrode is placed first in L2/3 and then in L4. Middle, Example of a pair of synaptic responses evoked in L2/3 and L4 in the same cell. Right, PPR of bipolar control cells when stimulated in L2/3 and L4 (*p* = 0.8, *t* = 0.20). Statistics: paired *t* test. *p < 0.05. ***p < 0.001.

To directly test the relationship between Prox1 and the expression of Elfn1 in multipolar and bipolar cells, we turned to a pharmacological agent, MSOP, which acts as an antagonist for presynaptic mGluRs (including mGluR7), which interact trans-synaptically with Elfn1 (Fig. 3*F*) (Tomioka et al., 2014). Application of MSOP indirectly tests for the presence of Elfn1 in control and Prox1 cKO VIP cells by blocking the constitutive suppression of synaptic release that Elfn1 induces through mGluRs (Stachniak et al., 2019). We therefore evoked excitatory synaptic events onto the two VIP subtypes as described above and assessed changes in neurotransmitter release in response to MSOP. We found that MSOP did not affect synaptic facilitation onto control (PPR: 1.11 ± 0.18 at baseline vs 1.04 ± 0.14 in MSOP) or Prox1 cKO VIP bipolar cells (PPR: 0.95 ± 0.29 at baseline vs 0.88 ± 0.24 in MSOP) (Fig. 3*G*,*H*). In contrast, MSOP markedly reduced the PPR of excitatory inputs onto control VIP multipolar cells (PPR: 1.43 ± 0.14 at baseline vs 1.11 ± 0.09 in MSOP) (Fig. 3*I*). However, the effect of MSOP was absent in the Prox1 cKO VIP multipolar cells (PPR: 1.37 ± 0.19 at baseline vs 1.23 ± 0.21 in MSOP) (Fig. 3*J*). These pharmacological results suggest that Elfn1/mGluR7 complexes are only present in VIP multipolar synapses and that, on Prox1 removal, the levels of these complexes decrease enough to be functionally undetectable. To test this selective distribution anatomically, we collected brains from control and Prox1 cKO animals (*VIPCre x Ai14 x Prox1fl*) at P21 and performed immunohistochemistry to label mGluR7-positive terminals. Presynaptic mGluR7 immunostaining is robust and requires the presence of postsynaptic Elfn1, thus providing an indirect measure of synaptic Elfn1 levels (Stachniak et al., 2019). Since bipolar cells have dendrites that extend over both L2/3 and L4, while multipolar cell dendrites tend to be restricted locally to L2/3, we reasoned that fluorescently labeled processes in L4 would stem predominantly from bipolar cells, while processes in L2 (upper L2/3) derive from both multipolar and bipolar neurons (Fig. 4*A*). We therefore quantified mGluR7 puncta colocalization with VIP processes in both upper L2/3 and L4 and compared between control and cKO conditions (Fig. 4*B*,*C*). First, we found that in control tissue L2 processes showed greater colocalization than those in L4 (Fig. 4*D*). Within L2, mGluR7 levels on VIP processes significantly decreased in Prox1 cKO compared with control condition (Fig. 4*E*). Since L4 processes (predominantly bipolar) showed no significant changes in mGluR7 levels on Prox1 removal (Fig. 4*F*), the large difference we see in L2 Prox1 cKO is likely driven by changes on multipolar processes. To separately test whether bipolar cells show laminar differences in mGluR7 levels, we elicited synaptic responses onto bipolar cells by first stimulating in L2/3 and then stimulating in L4 and measuring the PPR. Within-cell analysis showed no differences in PPR between the two layers (L2/3 mean PPR 1.03 ± 0.13, L4 mean PPR: 1.01 ± 0.12) (Fig. 4*G*), suggesting that the laminar differences we observe are indeed because of differences in VIP subtype mGluR7 and by proxy Elfn1 levels.

### Prox1 regulates *Elfn1* expression in all VIP INs

**Figure 5.**
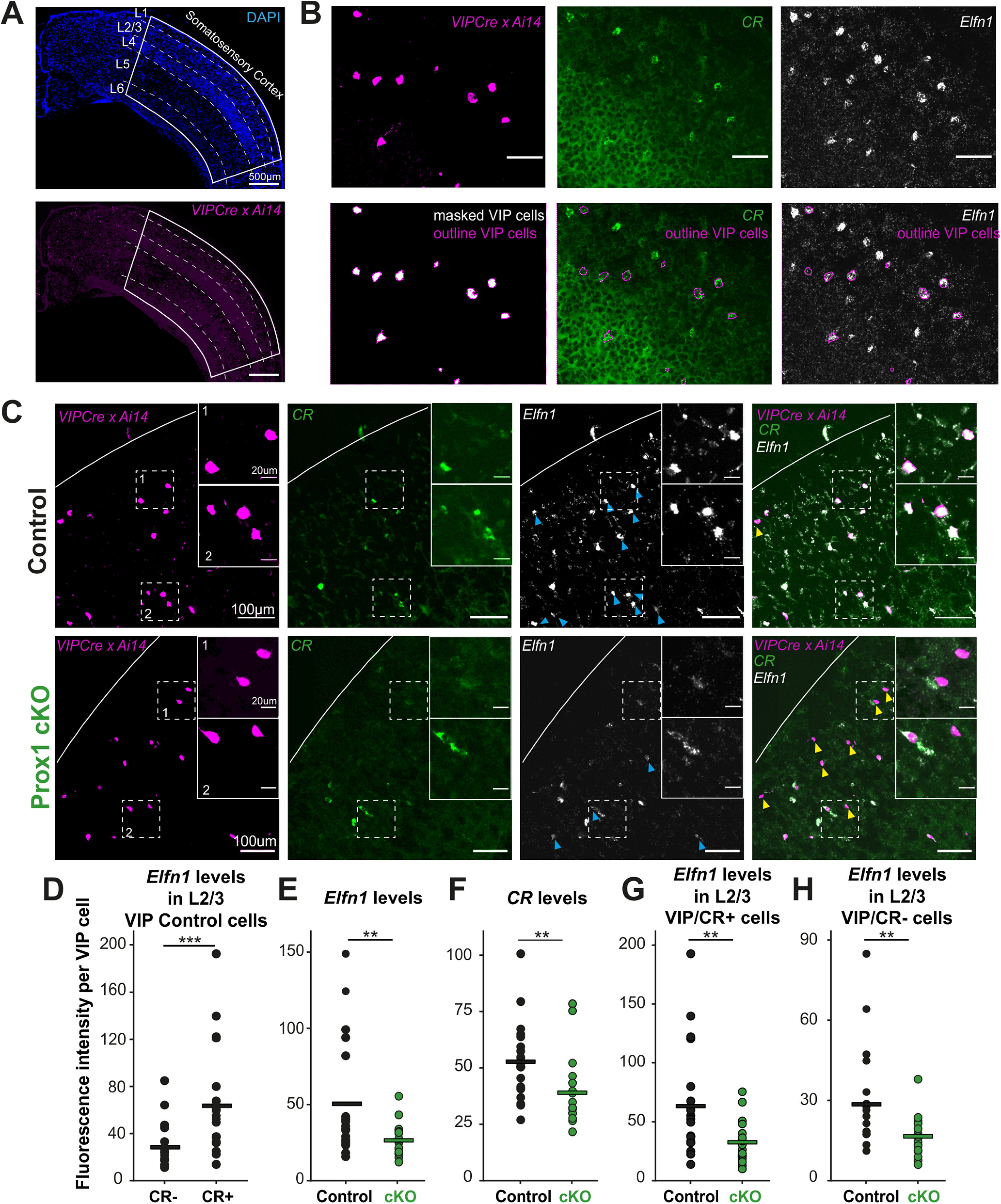
Prox1 regulates *Elfn1* expression in all VIP INs. ***A***, Manual segmentation of somatosensory cortex and layers 1-6 based on the DAPI channel of coronal sections. ***B***, An example image showcasing the high-throughput analysis method. VIP cells were segmented, and *CR* and *Elfn1* fluorescence levels were measured within the boundaries of the cell. ***C***, Examples of *Elfn1* and *CR* mRNA *in situ* (RNAscope) labeling in L2/3 tdTomato-positive VIP cells in control (top) and Prox1 cKO (bottom) tissue at P12. Insets, Close-up of the dashed-line boxes, including *CR*^+^ and *CR*^–^ VIP cells. White parabolic line indicates the pia. Yellow arrows highlight VIP-positive *Elfn1-*negative cells. Blue arrows highlight *Elfn1-*positive VIP cells. There are also *Elfn1-*positive cells that are not VIP-positive likely corresponding to SST cells. ***D***, *Elfn1* levels in VIP multipolar (*CR*^–^) and bipolar (*CR*^+^) cells in L2/3 of the somatosensory cortex (*p* = 0.001) (*N* = 3 animals, *n* = 19 sections/images). ***E***, *Elfn1* levels in VIP cells in all layers and cortical areas in control (*N*/*n* = 3/19) and Prox1 cKO (*N*/*n* = 2/18) tissue (*p* = 0.004). ***F***, *CR* levels in VIP cells in all layers and cortical areas in control (*N*/*n* = 3/19) and Prox1 cKO tissue (*N*/*n* = 2/18) (*p* = 0.004). ***G***, *Elfn1* levels in VIP bipolar cells in L2/3 of the somatosensory cortex in control (*N*/*n* = 3/19) and Prox1 cKO (*N*/*n* = 2/18) tissue (*p* = 0.004). ***H***, *Elfn1* levels in VIP multipolar cells in L2/3 of the somatosensory cortex in control (*N*/*n* = 3/19) and Prox1 cKO (*N*/*n* = 2/18) tissue (*p* = 0.006). Statistics: Mann–Whitney *U* test. **p < 0.01. ***p < 0.001.

Previous research has shown that both VIP bipolar and multipolar cells express *Elfn1* in the adult cortex (Paul et al., 2017; Tasic et al., 2018). These transcriptomic data stand in apparent contradiction to the VIP multipolar cell-selective effects we observe in our Prox1 loss of function, Elfn1 downregulation, and pharmacological experiments. We therefore hypothesized that either *Elfn1* is expressed only in multipolar cells before P21 or that Prox1 regulates *Elfn1* expression only in multipolar cells. To test these two possibilities, we collected and sectioned brains from Prox1 control and cKO mice (*Prox1fl* x *VIPCre* x *Ai14*) at P12 and performed ISH for *Elfn1* mRNA and *CR* mRNA to distinguish putative bipolar (*CR*^+^) and multipolar (*CR*^–^) VIP cells. We then obtained images of whole cortices and analyzed them using a custom-written MATLAB script (see Materials and Methods; Fig. 5*A–C*). We first assessed the presence of *Elfn1* mRNA in control VIP cells at P12 and found that, similarly to the adult cortex (Paul et al., 2017; Tasic et al., 2018), *CR*^+^ VIP cells express the gene at a higher level than *CR*^–^ VIP cells (Fig. 5*D*). This finding negates the first hypothesis regarding an age-dependent selective expression of *Elfn1* in multipolar cells.

We subsequently compared *Elfn1* and *CR* mRNA levels between control and cKO tissue and found a clear reduction of both signals (Fig. 5*E*,*F*), which is in line with the RNAseq results that showed a downregulation of both genes (log2 ratio of −1.466 for *CR* [*Calb2*] and −1.065 for *Elfn1*). Intriguingly, the reduction in *Elfn1* mRNA was seen for both *CR*^+^ (bipolar) VIP cells and *CR*^–^ VIP ones (most of which belong to the multipolar subtype) (Fig. 5*G*,*H*). These results suggest that Prox1 regulates *Elfn1* in both subtypes of VIP cells, negating our second hypothesis.

### Elfn1 overexpression changes the synaptic phenotype of VIP bipolar cells

**Figure 6.**
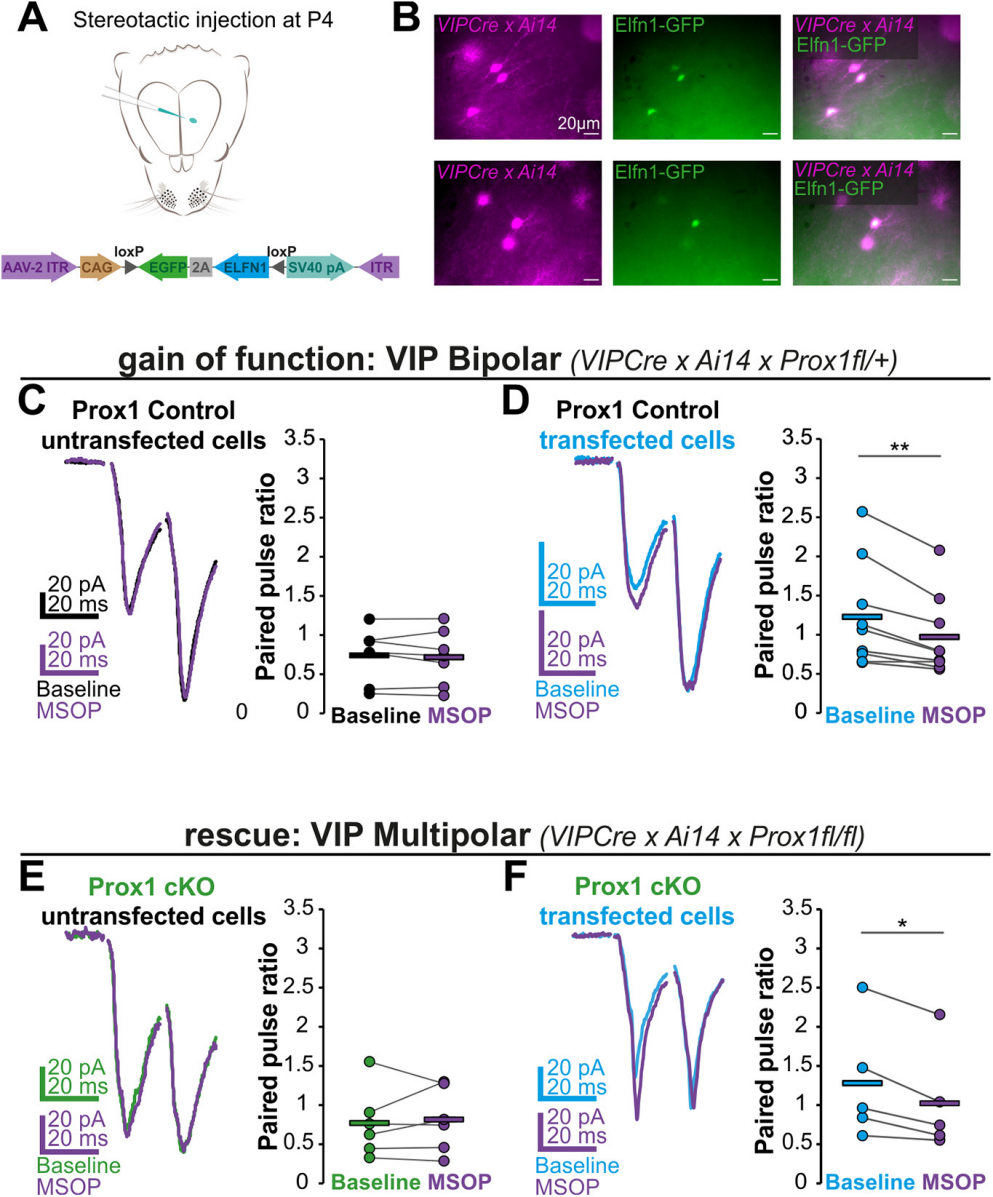
Elfn1 overexpression leads to synaptic facilitation in all VIP INs. ***A***, Schematic representation of the viral injections and the Elfn1-GFP plasmid. *VIPCre x Ai14 x Proxfl* control and cKO pups were injected at P4 with an AAV, which was left to integrate for 2-4 weeks. ***B***, Example images of Elfn1-GFP transfected VIP bipolar (top) and multipolar (bottom) cells as seen under the electrophysiology microscope. ***C-F***, Left, Example of a pair of synaptic responses evoked by stimulation (scaled to second evoked response). Right, All data points for PPR are plotted. ***C***, PPR of bipolar Prox1 control untransfected cells (*n*/*N* = 7/6) under baseline conditions and with MSOP (*p* = 0.5, *t* = 0.73). ***D***, PPR of bipolar Prox1 control Elfn1-GFP transfected cells (*n*/*N* = 9/7) under baseline conditions and with MSOP (*p* = 0.004, *t* = 4.07). ***E***, PPR of multipolar Prox1 cKO untransfected cells (*n*/*N* = 6/5) under baseline conditions and with MSOP (*p* = 0.7, *t* = 0.46). ***F***, PPR of multipolar Prox1 cKO Elfn1-GFP transfected cells (*n*/*N* = 5/4) under baseline conditions and with MSOP (*p* = 0.02, *t* = 4.00). Statistics: paired *t* test. *p < 0.05, **p < 0.01.

A potential explanation for the discrepancy between *Elfn1* transcript levels and function in VIP bipolar cells is that Elfn1 protein is largely absent from the synapse, either because the mRNA is not translated or because the protein is not transported there efficiently. We therefore examined the cell-autonomous effects of overexpressing Elfn1 protein in VIP bipolar neurons. We induced sparse virus-based overexpression of Elfn1 along with GFP in 3- to 5-week-old control bipolar cells and assessed the resulting short-term dynamics of synaptic responses, as well as the effect of MSOP on transfected and neighboring untransfected cells (Fig. 6*A*,*B*). As before, we found that, in untransfected VIP bipolar control cells, MSOP did not affect PPR (PPR: 0.73 ± 0.13 at baseline vs 0.72 ± 0.13 in MSOP) (Fig. 6*C*). In contrast, when the bipolar cells overexpressed Elfn1, the PPR of excitatory inputs increased and MSOP significantly reduced it (PPR: 1.23 ± 0.22 at baseline vs 0.97 ± 0.17 in MSOP) (Fig. 6*D*). These results suggest that the absence of Elfn1 function in bipolar cells is indeed because of a lack of protein at the synapse, which can be overcome by overexpression of Elfn1.

In parallel experiments, we also assessed the overexpression of Elfn1 in 3- to 5-week-old multipolar VIP Prox1 cKO cells to see whether we can rescue the synaptic phenotype. In untransfected Prox1 cKO multipolar cells, we found that MSOP did not affect PPR (PPR: 0.77 ± 0.18 at baseline vs 0.81 ± 0.17 in MSOP) (Fig. 6*E*), while it significantly reduced PPR in transfected neurons (PPR: 1.28 ± 0.34 at baseline vs 1.02 ± 0.30 in MSOP) (Fig. 6*F*). Thus, by reintroducing Elfn1 into Prox1 cKO multipolar cells, the phenotype can be rescued to resemble the control condition.

## Discussion

In the mammalian nervous system, the TF Prox1 is known to regulate cell-cycle exit (Cid et al., 2010; Kaltezioti et al., 2010), cell fate determination (Iwano et al., 2012; Kaltezioti et al., 2014) of neural precursor cells, and neurite outgrowth (Kaltezioti et al., 2021). Prenatal removal of Prox1 in embryonic caudal ganglionic eminence-derived cortical INs results in a failure of CR^+^ VIP INs to migrate to the correct cortical layer, a significant decrease in their numbers, and a subsequent reduction in the excitatory synaptic input onto the remaining cells (Miyoshi et al., 2015). Postnatal removal of Prox1 circumvents cell death and layer mis-targeting. Nevertheless, we find a continued requirement for the TF in the regulation of synaptic dynamics and the final specification of VIP subtypes. During the first two postnatal weeks, when VIP INs are embedding into their circuits, Prox1 upregulates genes related to synaptic processes and neuronal cell fate. Cell-specific expression of synapse-related genes, such as cell adhesion molecules, help guide the cell–cell interactions that ultimately determine the connectivity, morphologic, and electrophysiological characteristics of IN subtypes (Paul et al., 2017; Gouwens et al., 2020). One of these synapse-related genes is *Elfn1*, which codes for a trans-synaptic protein that produces synaptic facilitation of excitatory inputs in multipolar VIP cells. Although Prox1 also regulates the expression of *Elfn1* transcript in bipolar VIP cells, they show negligible facilitation and Prox1 removal does not impact bipolar PPR. Thus, by regulating VIP subtype-specific Elfn1 engagement, the TF Prox1 promotes and maintains functional diversity in VIP IN subtypes.

Prox1-dependent upregulation of Elfn1 leads to excitatory synaptic facilitation specifically in VIP multipolar cells. In SST INs, this Elfn1-dependent facilitation of excitatory inputs prevents rapid recruitment, thereby creating their characteristic “delayed” firing property (Sylwestrak and Ghosh, 2012) and biasing responsiveness toward high-frequency activity (>20 Hz) (Pouille and Scanziani, 2004; Stachniak et al., 2019). The same mechanism would allow VIP multipolar cells to selectively tune to the high-frequency activity characteristic of corticocortical communication, whereas bipolar neurons would preferentially tune to lower excitation frequency (4-12 Hz) (Palmer et al., 2012; Luo et al., 2020). Within L2/3, VIP multipolar cells are often found close to the border with L1, whereas VIP bipolar cells usually are located closer to L4, which, in combination with their distinct dendritic architecture, could bias the amount of thalamocortical and corticocortical inputs received by the VIP subtypes (He et al., 2016; Sohn et al., 2016). Thus, by regulating Elfn1, Prox1 may prime multipolar neurons to coordinate intracortical communication within the superficial layers of the cortex. Interestingly, the SST INs do not express Prox1; therefore, our results not only identify a novel functional role for Elfn1 in VIP multipolar INs, but also reveal a novel regulatory pathway for *Elfn1* expression in general.

In contrast, short-term plasticity of excitatory inputs onto bipolar cells is unaffected by removal of Prox1, by decreased *Elfn1* expression, or by the pharmacological blockade of its synaptic effects. These findings were very surprising given that our results and published transcriptomic data show high levels of *Elfn1* mRNA expression in bipolar VIP cells. Thus, it appears that *Elfn1* mRNA levels are decoupled from Elfn1 function in this VIP subtype. Noting that mGluR7 protein levels were consistent with our observations of subtype-specific Elfn1 function, we tested whether bipolar cells might have insufficient Elfn1 protein levels, despite the high mRNA level. Overexpressing the Elfn1 protein in bipolar VIP cells by viral injection led to the recruitment of mGluR7 to the presynapse, confirming that Elfn1 levels in VIP INs are the primary determinant of the synaptic phenotype, as found previously (Sylwestrak and Ghosh, 2012). Hence, our results indicate that Prox1 regulates the levels of *Elfn1* mRNA in both VIP subtypes, but that there is an additional mechanism present in bipolar cells that prevents Elfn1 protein from being produced. Possible mechanisms could include regulation of translation by microRNAs or alternative splicing. Indeed, it has been reported that *Elfn1* can be regulated by the *mirg* miRNA cluster in an induced neuronal culture system (Whipple et al., 2020). Irrespective of the exact mechanism, our experiments show that viral-induced overexpression of Elfn1 can override any post-transcriptional regulatory process that limits Elfn1 protein levels in bipolar cells. These findings suggest that subtype-specific post-transcriptional mechanisms contribute to VIP IN subtype diversity by reducing synaptic Elfn1 protein in bipolar cells.

In addition to the potential post-transcriptional regulatory mechanisms influencing Elfn1 protein levels, VIP subtype-specific protein–protein interactions across the synapse may also exist. Indeed, Elfn1 has been shown to interact with multiple mGluR subtypes, and heterodimerization of mGluR7 with other mGluRs changes synaptic facilitation and MSOP responsiveness (Kammermeier, 2015; Levitz et al., 2016; Dunn et al., 2018). Homodimerization of mGluR7 produces constitutive activation of the receptor, conferring facilitating properties and MSOP sensitivity onto synapses (Kammermeier, 2015; Stachniak et al., 2019). In contrast, heterodimerization (e.g., mGluR7/mGluR2 or mGluR7/mGluR4) increases glutamate responsiveness, thus producing synaptic depression, and also leads to the loss of MSOP responsiveness (Kammermeier, 2015; Levitz et al., 2016; Stachniak et al., 2019). Thus in multipolar VIP synapses, higher levels of Elfn1 protein may trigger a cell-selective switch in PPR by promoting formation of mGluR7 homodimers over heterodimers.

The final specification of neurons is a process that takes place long after their birth, as they start incorporating themselves into the resident circuit. It is during this time of establishing connections that the needs of the circuit, as defined by those of the animal, can instruct a neuron toward its mature state, which includes the proper construction and function of inputs and outputs. For inhibitory INs, these input/output specificities vary considerably, not only between cardinal IN classes, but also between the subtypes within a class (Huang and Paul, 2019). We find that postnatal Prox1 expression in VIP cells diversifies the dynamics of incoming excitation onto the multipolar versus the bipolar subtypes. Our rescue and overexpression experiments in late juvenile/young adult mice (postnatal weeks 3-5) show that Elfn1 expression is necessary to maintain this synaptic diversity. Since Prox1 is present in VIP INs of adult mice, it is likely that the continued presence of Prox1 is required for maintaining the synaptic phenotype we have uncovered. Our data provide evidence that the continuous expression of the TF Prox1 is necessary for the final specification of bipolar and multipolar VIP cells, allowing them to acquire diverging roles within the adult cortical network.
